# The Chronic Care Model and Technological Research and Innovation: A Scoping Review at the Crossroads

**DOI:** 10.2196/jmir.3547

**Published:** 2015-02-06

**Authors:** Deede Gammon, Gro Karine Rosvold Berntsen, Absera Teshome Koricho, Karin Sygna, Cornelia Ruland

**Affiliations:** ^1^Norwegian Center for Integrated Care and TelemedicineUniversity Hospital of North NorwayTromsoeNorway; ^2^Center for Shared Decision Making and Collaborative Care ResearchOslo University HospitalOsloNorway; ^3^The National Research Center in Complementary and Alternative MedicineUniversity of TromsøTromsøNorway

**Keywords:** chronic care model, chronic disease management, information and communication technology, telemedicine, ehealth, computer science, medical informatics, scoping review

## Abstract

**Background:**

Information and communication technologies (ICT) are key to optimizing the outcomes of the Chronic Care Model (CCM), currently acknowledged as the best synthesis of available evidence for chronic illness prevention and management. At the same time, CCM can offer a needed framework for increasing the relevance and feasibility of ICT innovation and research in health care. Little is known about how and to what extent CCM and ICT research inform each other to leverage mutual strengths. The current study examines: What characterizes work being done at the crossroads of CCM and ICT research and innovation?

**Objective:**

Our aim is identify the gaps and potential that lie between the research domains CCM and ICT, thus enabling more substantive questions and opportunities for accelerating improvements in ICT-supported chronic care.

**Methods:**

Using a scoping study approach, we developed a search strategy applied to medical and technical databases resulting in 1054 titles and abstracts that address CCM and ICT. After iteratively adapting our inclusion/exclusion criteria to balance between breadth and feasibility, 26 publications from 20 studies were found to fulfill our criteria. Following initial coding of each article according to predefined categories (eg, type of article, CCM component, ICT, health issue), a 1st level analysis was conducted resulting in a broad range of categories. These were gradually reduced by constantly comparing them for underlying commonalities and discrepancies.

**Results:**

None of the studies included were from technical databases and interventions relied mostly on “old-fashioned” technologies. Technologies supporting “productive interactions” were often one-way (provider to patient), and it was sometimes difficult to decipher how CCM was guiding intervention design. In particular, the major focus on ICT to support providers did not appear unique to the challenges of chronic care. Challenges in facilitating CCM components through ICT included poorly designed user interfaces, digital divide issues, and lack of integration with existing infrastructure.

**Conclusions:**

The CCM is a highly influential guide for health care development, which recognizes the need for alignment of system tools such as ICT. Yet, there seem to be alarmingly few touch points between the subject fields of “health service development” and “ICT-innovation”. Bridging these gaps needs explicit and urgent attention as the synergies between these domains have enormous potential. Policy makers and funding agencies need to facilitate the joining of forces between high-tech innovative expertise and experts in the chronic care system redesign that is required for tackling the current epidemic of long-term multiple conditions.

## Introduction

**Figure 1:**
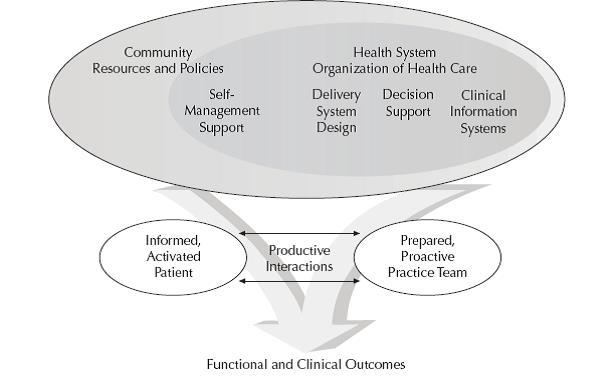
The Chronic Care Model. (Reprinted with permission from American College of Physicians).

One of the biggest health care challenges worldwide is the growing number of persons with chronic or lifestyle-related illness, which is threatening the infrastructure of health care systems by rising demands and unsustainable costs [1]. Today’s fragmented service delivery between levels of care is partly blamed for the escalation of health care costs seen internationally. The Chronic Care Model (CCM) is acknowledged currently as the best synthesis of available evidence for chronic illness prevention and management interventions [2,3] (see Figure 1). Since launched by Wagner and colleagues in the late 1990s [4,5], the model has been extensively elaborated and expanded upon, for example, by the World Health Organization to highlight macro issues related to population health and health promotion [6,7].

Nevertheless, the basic components of the original CCM remain core to modern chronic care system redesign of clinical practices. The model comprises six components, each of which are supported by evidence as contributing toward productive patient-provider interactions and improved outcomes.

While questions still remain about whether sequential versus full implementation of the components are associated with differences in outcomes [2], orchestration of the six components are assessed in terms of how well they support productive interactions between the informed, activated patient and the prepared proactive practice team. Key to the model is an acknowledgement of the patient’s own role in self-management as a vital, but under-focused, resource in chronic care. This entails a fundamental shift for health care that is traditionally built around acute, episodic encounters. Long-term and individualized support for self-management, in partnership with a proactive (rather than reactive) multi-professional team, is thus a central feature of this model and the evidence that supports it [8].

Information and communication technologies (ICT) are becoming ubiquitous to the information infrastructure of health care. While the CCM-component “clinical information systems” (electronic medical records, disease registries) is by definition ICT-based, several call for increased use of ICT to facilitate implementation and fidelity of the other CCM-components [9,10]. Advancements in the technological domains of computer science and information technology are fast-paced, as indicated by the last 10-20 years of high-tech products that have altered everyday life in Western civilization. Indeed, the market of direct-to-consumer mobile health and wellness products and apps is estimated to reach US $26 billion globally by 2017 [11]. Similar developments are gaining momentum under headings such as “assisted or independent living” and “welfare technologies” [12], many of which are potentially well-suited for patient-centric solutions within a CCM framework.

Nevertheless, similar to the gap between medical evidence and practice [13], there is a gap between technological research and innovation, and applications in health care. This is evident in that telemedicine and eHealth systems with documented benefits often fail to become incorporated into routine clinical practice [14]. Explanations offered include a mismatch between accepted methods in medicine (eg, randomized controlled trials) and the socio-technical nature of ICT systems, as well as a neglect in medical informatics and telemedicine to articulate theoretical rationales for the systems they design and expected outcomes. This undermines an ability to communicate between stakeholders, prioritize innovations, sort out critical variables in adapting them, and explain successful and unsuccessful outcomes [15]. Others note a lack of attention to contextual issues during implementation [14]. Thus, while many ICT innovations may be well-suited for facilitating CCM, they often end as pilots, detached from the broader movement toward improving chronic care in line with available evidence.

Arguably, CCM represents a type of framework that can aid in increasing the relevance and implementation of technological research and innovation to health care. First, it is comprehensive as well as intuitive, thus enabling a common language that may bridge the communication difficulties between health care stakeholders (patients, providers, funders) and technologists. Second, often framed as quality improvement, CCM can be linked to approaches that health care professionals are increasingly acquainted with (quality collaborative, breakthrough methodologies) and that are well-suited for ICT implementations [16-18]. Third, as the evidence-base of CCM increases, an increasing number of national and regional health care organizations are redesigning their health care services in accordance with CCM [2]. This provides a broader and more cohesive framework for the piloting and implementation of large-scale trials of innovative ICT applications. Further, while some ICT applications may only target one or two CCM components, adherence to the CCM framework should nevertheless enable better integration between applications supporting the other components.

These observations led us to examine the state of work being done at the crossroads of CCM and ICT research domains by examining how ICT is used to support the six domains of the CCM. Our overall motivation is identify the gaps and potential that lie between these research domains, thus enabling more substantive questions and opportunities for accelerating improvements in ICT-supported chronic care.

## Methods

### Study Design

A scoping study approach is a type of review that helps rapidly identify gaps in existing literature and points out areas worth further attention [19,20]. We initially considered conducting a broader scope of the chronic care literature than CCM. It became readily apparent, however, that inclusion of related concepts (chronic care, integrated care, coordinated care, disease management, shared decision making) resulted in a magnitude of literature that was unlikely to offer the types of insights we were seeking, even if we had the resources to analyze it conscientiously. This included extensions of CCM such as that of the World Health Organization, which emphasizes public health and health promotion in communities [6]. Our specific interest in clinical system redesign, coupled with the above arguments about the role of models such as CCM in facilitating stakeholder communication, led us to limit our focus to the basic CCM components. The process of determining inclusion and exclusion criteria was a team process that evolved iteratively during the initial broad searches of key concepts.

**Figure 2:**
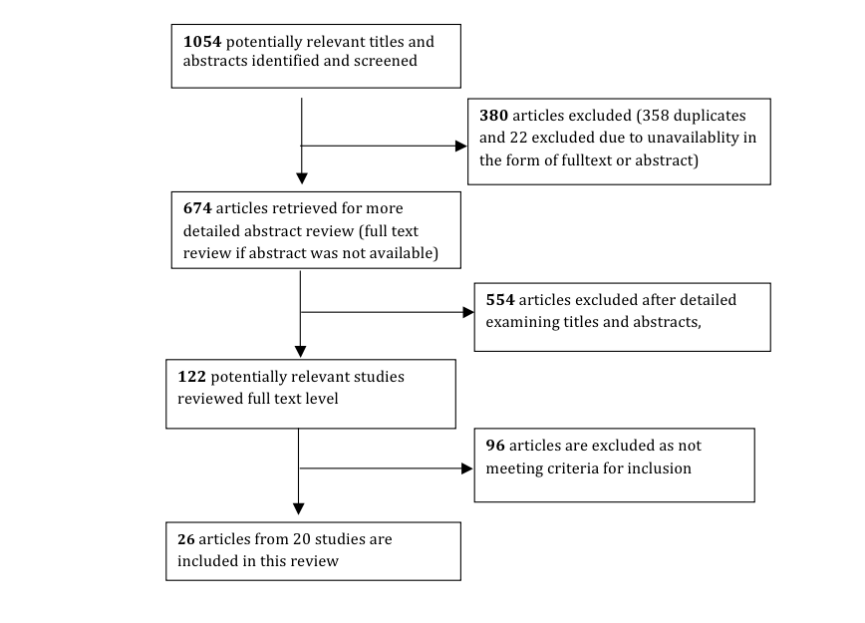
Flow chart of search results.

The following inclusion and exclusion criteria were applied. Inclusion criteria were: (1) a general focus that is apparent in the abstract on both CCM-theory/ implementation/ practice within a health care setting, and ICT-research and innovation, including innovative use of mature ICT-tools, with a purpose of supporting CCM-practice, and (2) any type of study (review, field study, theoretical analysis, randomized controlled trials). Exclusion criteria were: (1) papers where the CCM or ICT innovation was only peripherally mentioned and was not integral to the main focus of the paper, (2) protocols or abstracts not followed by a peer-reviewed full text publication, (3) commentaries, editorials, letters, and (4) technical feasibility trials. (See Figure 2.)

### Information Sources and Search Strategy

The “Chronic Care Model” was used as the main search term combined with ICT. However, similar to what Coleman found [2], variations in nomenclature used by authors and imprecise descriptions of interventions made it difficult to meaningfully identify CCM-based interventions. Thus, to facilitate the search for and collection of relevant articles, we used the Science Citation Index-Web of Knowledge search tool to gather articles that cite one of five seminal articles [4,21-24] that together originally described the CCM.

In other databases, we searched for English-language publications in a selection of medical/health (Medline, Embase, PsycINFO, Cinahl, and Cochrane Library) and technical (IEEE, ACM Digital Libraries) databases. In the medical/health databases, any paper that included a title, abstract, or keyword referring to ICT-synonyms was considered to be a health ICT-paper. In technical databases, we considered the subset of papers with a health focus to be health ICT-papers either including health-ICT synonyms (eg, health informatics, telemedicine), subject headings, title, or abstract such as “telemedicine”, or papers including a “health” synonym. See Multimedia Appendix 1 for a complete list of search terms. The first search was conducted in October 2011, and last updated in October 2013.

### Review of Eligible Papers

Search results were exported to EndNote (X6) (Thomson Reuters, Carlsbad, CA, USA) for merging of databases, identification and deletion of duplicates, and review management. Papers that were identified by database search algorithms as belonging to both the CCM and the health-ICT domain were collected in one common reference database for all CCM and health ICT-papers. In all, 1054 references were identified in all eight databases, of which 358 were identified as duplicates, and 22 excluded due to unavailability in the form of an abstract or full text, leaving 674 unique references eligible for abstract evaluation. In line with scoping studies [19], inclusion was not restricted to specific types of studies (eg, qualitative and quantitative), participants, types of intervention, or type of outcome.

A total of 122 articles were compliant with the above criteria and retrieved in full text for evaluation of eligibility.

### Study Selection and Data Collection Process

If the publication did not have an abstract or the abstract was unclear with respect to the degree of CCM focus, the full text was retrieved. Otherwise, eligibility of all papers was primarily based on abstract evaluation.

The validity and reliability of the above inclusion/exclusion criteria was tested in a subset of 40 full-text references that were reviewed both in abstract and in full text, independently by two authors (DG/GB). Of 40 papers, both DG and GB agreed on inclusion of nine papers, exclusion of 30 papers, and disagreed on one paper. Further evaluation of inclusions were done by GB alone, and discussed with DG in cases of ambiguity. The two authors (ATK and KS) extracted data based on the inclusion and exclusion criteria into a structured spreadsheet. All disagreements were resolved by consensus discussion and four articles were discussed in a mini workshop by four authors (ATK, GB, KS, DG) for the final inclusion decision.

When authors reported primary and secondary analyses from the same study in two separate articles, we present them as one study and two articles.

### Data Extraction and Management

Authors ATK or KS extracted the following variables from each included article: (1) eligibility criteria, (2) study design, (3) methodology, (4) target groups and topics, (5) the type of ICT used, (6) how the study integrated CCM and all its components, (7) the scale of the implementation, and (8) outcome measures and results relevant to the CCM-ICT implementation.

### Analysis Process

In line with scoping studies and the aim of this study, we combined quantitative and qualitative analysis of selected articles resulting in both a descriptive numerical summary and a thematic analysis [19]. Predefined descriptive categories were applied to the initial coding of all articles: to type of article (eg, conceptual and intervention study), topic (eg, disease, technology, patient, provider, both), and issues addressed. All included articles were then examined by ATK and KS using a qualitative content analysis approach [25] resulting in a broad range of dimensions and categories. These were gradually reduced by constantly comparing them for underlying commonalities and discrepancies. Analysis notes and emerging categories were linked to the articles and concepts supporting each category. This allowed co-authors (DG, KS, GB, and CR) to discuss categories and alternative descriptions, although this was only done when co-authors did not intuitively understand the proposed categories. Any disagreement between the reviewers was resolved by consensus discussions.

## Results

### Descriptive Numerical Summary

The 26 included publications described 20 different studies, all of which were from medical databases. A total of 80% (16/20) of the studies were conducted in the United States, and the rest of the studies were from The Netherlands (n=1), Italy (n=1), Cyprus (n=1), and one study from six Asian countries. Eight studies had been published between 2004 and 2008 and 12 between 2009 and October 2013.

Of the total 20 included studies, 14 used quantitative methodology, four used qualitative methodology, and two studies combined quantitative and qualitative methodologies.

Patient groups were all defined by their health conditions. Diabetes was by far the most common disease type targeted by ICT-CCM implementation studies and accounts for 10 studies of the 20 included studies.

The summary of studies and the diseases that they looked at, the components of the CCM they focused on, and the type of ICT intervention they implemented are presented in Multimedia Appendix 2.

### Presentation and Discussion of Thematic Analysis

#### ICT to Support Patient-Provider Interaction

“Productive interactions” is a critical dimension of CCM and thus of particular interest in this study. A total of 13 out of the 20 papers had ICT-CCM interventions that supported communication between patients and health care providers. Seven of these were one way (from providers to patients), while six offered patients the option to communicate with their providers using the designated ICT. However, for the most part, patients were involved by submitting predefined measures such as signs and symptoms, that is, providing clinical decision support for clinicians and/or patients.

Emails or text messages using mobile phones, secure Web-based systems, and telephone lines were found in 62% (8/13) of the studies that had a primary focus on patient-provider communication [26-33]. This included secure communications that also allowed patients to have full [30] or partial [27,29] access to their electronic health records (EHRs). Additional support included functions such as preventive health reminders, disease-specific information, self-care advice as a response to symptoms and test results, medication refill, appointment booking, laboratory test results, clinic visit summaries, lists of allergies, immunization status, and biometrics [27,31,34]. (See Multimedia Appendix 2.)

The second most common means of patient-provider communications were telephones (n=5), which we included to capture usage of mobile phones. However, only one study [30] used smartphones, two studies used text messaging [32,33], one used analogue telephone lines to transfer data [26], while three used ordinary voice telephony [35-37]. In some of these studies, telephone calls were the only means health care providers had to reach their patients [34-36]. This was done to remind patients when their tests were overdue [34], to provide self-management support to patients using either computer-assisted health education scripts [35], or as scheduled weekly calls to support self-management [36].

Video technology was only used in the Darkins and colleagues [26] study, and only as a tool to support patients needing assistance on how to use their other communication devices and biometric devices to send data to their health care providers. It was reported that it was hardly used. Finally, fax was used for daily data feeds from independent laboratories and automatic test interpretations were sent by fax and mail to providers and patients if not easily reached by electronic networks [31].

In light of the importance CCM places on “productive interactions” and facilitating self-management, it is somewhat surprising that so few studies (six) in our sample appear to leverage ICT for this purpose. Seven of the studies had one-way communication (provider to patient) without offering rationales. The degree to which patients actually were engaged in the management of their care is apparently assumed, but little illuminated. 

#### ICT for Providers Across CCM Components 

Seven of the 20 papers primarily focused on communication between health care providers and/or targeted the “clinical decision-making” component. Interventions in this category included physician education and feedback to physicians [38], provider feedback with guideline-driven medication assistance prompts [39], Web-based clinical decision support for providers [40], specialist and primary care physician email communication [41], and secure communication between psychiatric care team composed of primary care physicians, psychiatrists, and supporting nurses [42]. Other similar interventions included a Web-based decision support program that also provided feedback report to patients [43], a Web-based decision support system [40,42], and a Web-based feedback to clinicians with a simultaneous feedback report system for patients [39]. Implementation of EMRs and computerized disease registries to help support clinical data collection [24,38,42] were also among the ICT interventions.

While clinical decision support and effective provider-provider communication are vital components in CCM, it was often difficult to decipher how the interventions were expected to more specifically contribute either to the “informed activated patient”, the “prepared proactive team”, or both. Further, descriptions of how the interventions were expected to interact with, or at least complement, other CCM-components such as “self-management support” or “delivery system design” were typically lacking. In these cases, it was not apparent why CCM was used as a framework at all.

#### The CCM − ICT Gap

The ICTs in the included studies can be characterized as “old-fashioned” (with the exception of an unsuccessful experiment with gaming technology [30]). None of the studies in our sample were published in technical research venues. This apparent neglect of ICT research and innovation to embrace state-of-the-art approaches to solutions for chronic care is worth noting and may reflect a number of factors.

First, ICT innovations that are introduced into health care typically need to interact with pre-existing, often highly complex and inflexible systems, such as EHR. Testing ICT innovations in real-life clinical practices, even “simple” plug-in interventions, often require developing interfaces with EHR systems, which in itself can be costly and complex both legally and organizationally. This may discourage decision-makers in health care organizations from embarking on innovation processes. Technologists on the other hand need expeditious environments where they can iteratively test and evolve innovations before market deployment.

Second, ICT research faces the same type of “translational” challenge as medical research. ICT research typically tests “hypotheses” through prototypes which, as with medical research findings, often fail to translate into contexts of practice [44]. It can be argued that many ICTs could be well suited for solutions in chronic care, had broader frameworks (eg, CCM) been used to facilitate the multidisciplinary and stakeholder dialogue necessary for adapting and applying innovative solutions to contexts of practice.

Third, ICT-interventions involving patients face digital divide issues related to accessibility regardless of income and digital literacy. Important work in addressing this challenge is found in ICT research and innovation explicitly targeting elderly populations, and is often referred to as “independent or assisted-living technologies” [45] and “welfare technologies” [12]. Inspired by disability research, these domains more explicitly adhere to universal design principles and low-cost accessibility. We were somewhat surprised that our study did not detect any work from this area, possibly reflecting sectorial distinctions between health care (from which CCM emerged) and disability/social services (from which welfare technologies emerged).

It would be worthwhile to explore more closely the causes of the apparent gap between CCM and ICT innovation, as well as the potential of CCM to facilitate productive synergies with work being conducted on welfare technologies.

#### CCM Lost in Translation

In most of the articles in this review, authors start by describing thoroughly all the components of the CCM and how important it is to integrate them in their upcoming implementation. However, there was a tendency to restrict the interventions to selected CCM components during the course of the implementation process. For example, Samoutis and colleagues [46] discussed all the components of the CCM in the planning phase, but dropped self-management support and utilization of community resources during the intervention, without offering rationales. Some explicitly limited their focus to certain components of the CCM, while the study by Darkins and colleagues was the only study where ICT interventions supported more or less all six components of the CCM [26].

The CCM components most focused upon in our included studies were delivery system design, decision support, and self-management support. The CCM components that were least associated with ICT implementation were community resources and health system organization. While the first is an obvious candidate for facilitation through social media, none of the studies reviewed suggested this.

Inconsistency in the integration and application of the CCM components was observed throughout our sample. Almost none of the CCM-ICT interventions that we have included are alike, or follow the same pattern of implementation. Also, CCM’s basic principles of patient engagement, that is, shared decision making toward a care plan aligned with patient needs, values, and preferences were barely detected in our sample.

These observations probably reflect the nature of CCM. It is an overarching framework for entire health care system design. To be useful, it needs to be operationalized and tailored to local context. This process has no guidelines. We see that the dual focus on the two main components (patients and teams) is often lost in this process.

CCM’s strength is its general and overarching focus on all system components, which has inspired health care reforms across the world. We have identified an important gap between the agenda of health care and the agenda of ICI research domains. The ICT world does not seem to know or understand the language and challenges represented by the CCM. Equally, the CCM champions do not seem to be aware of or capable of applying novel technologies in their approaches. 

#### Organizational and ICT Challenges

While few of the studies offered details about challenges, we noted the following: only a few studies managed to fund the interventions after the research/pilot projects ended [35,47]. Handing over the programs to non-profit managed care organizations was found to be one solution to sustain the programs [35]. Challenges also included provider resistance to using secure electronic messaging [29], along with challenges with the ICT itself, which ranged from minor technical problems [28] to absence of ICT resources (eg, computers, patient websites, and medical records) for successful integration of CCM-ICT interventions [27,24,47].

The lack of access to, for example, the Internet was also mentioned as a challenge, particularly for patients with low socioeconomic status or old age [27]. Individuals who are uninsured or publicly insured or those with communication barriers with limited literacy or limited language proficiency were also seen to be challenged by traditional mobile text messaging [36]. Similarly, use of unfamiliar ICT for patients and non-age appropriate ICT caused intermittent technical difficulties in uploading self-monitored blood glucose values [30]. Innovations to lessen the digital divide should be a major concern for further policies in chronic care.

## Discussion

### Principal Findings

This scoping study offers insights into the state of work being done at the crossroads of CCM and ICT with the intention of pinpointing possible gaps and synergies. The following is worth noting from this study.

The identified gap between CCM-inspired policy reforms and research and ICT research and innovation gives rise to important questions. What significant synergies can be leveraged by explicitly linking ICT research and innovation to CCM-based interventions? For example, what can the ICT research domain of Computer Supported Cooperative Work contribute in enhancing CCM’s productive interactions between patients and proactive care teams? The introduction of patients into cooperative work processes raises a range of issues that are both exciting and potentially of enormous impact. Exploring this potential would be worthwhile.

ICT innovations championed under headings such as “assisted living” and “welfare technologies” would seem well suited in supporting informed and active patients, and linking them to proactive care teams. Arguably, this would also help address digital divide issues noted in this review. Nevertheless, our study did not detect that this is happening. Rather, much of the work can be characterized by traditional medical informatics that supports the clinical work of providers.

Existing ICT infrastructures in health care (eg, EMR, data security issues) and the complexities, costs, and risks involved in changing them probably represent major barriers to innovation. It is perhaps not a coincidence that the Darkins study from the Veterans Administration was the most comprehensive both in terms of CCM and ICT. For health system organizations built around separate administrative levels of care, the complexities of negotiating innovative models of care across entities are even greater. Establishing large-scale living labs [48] or intermediate platforms for research and innovation that can safely interact with existing systems without disrupting ordinary clinical services may be one way of facilitating iterative innovation processes.

CCM offers a framework to aid communication across research domains and stakeholders. Other frameworks (eg, Patient-Centered Medical Homes [49]) can serve the same purpose assuming that they are supported by evidence and can facilitate communication between research domains and stakeholders. Given the complexities of chronic care, and the enormity of efforts needed to improve it, common frameworks such as CCM can increase the likelihood that the multitude of projects and innovations can be more systematically applied and assessed in terms of how well they contribute to improving the overall care delivered.

### Strengths and Limitations

Limiting our search to CCM is both a strength and weakness. The obvious weakness is that relevant work using similar models and concepts referred to in the chronic care literature are not included in the study. Thus, we cannot claim to offer a total overview of what is happening at the crossroads of chronic care and ICT research and innovation. The strength of limiting our search to CCM is that it is clearly defined, it is currently recognized as the best synthesis of evidence, and it serves as a framework for health system redesign in Western countries [2,3]. Also, most other system models for chronic care build on, or are an adaptation of, the CCM in some way. Thus, we are confident that our observations are relevant and worth attention also for those applying other chronic care frameworks. Another strength of this study is the novel approach to identifying synergies between domains of chronic care and ICT research and innovation. Identifying gaps and synergies is an important step in leveraging the resources of these domains to meet the massive challenges of chronic and lifestyle-related diseases.

### Conclusions

Efforts to bridge the gaps identified in this study need explicit and urgent attention as the synergies between domains of research have enormous potential. Policy makers, journals in the health-ICT field, and funding agencies need to facilitate the joining of forces between high-tech innovative expertise and experts in chronic care health system redesign that is required for tackling the epidemic of long-term multiple conditions in populations.

## Multimedia Appendix 1

Search terms.

## Multimedia Appendix 2

Included articles.

## Abbreviations

CCM: Chronic Care Model
EHR: electronic health record
EMR: electronic medical record
ICT: information communication technology

## References

[1] (2005). Preventing chronic diseases: a vital investment.

[2] Coleman K, Austin BT, Brach C, Wagner EH (2009). Evidence on the Chronic Care Model in the new millennium. Health Aff (Millwood).

[3] Oxman A, Bjørndal A, Flottorp S, Lewin A, Lindahl AK (2008). Integrated health care for people with chronic conditions: a policy brief.

[4] Wagner EH, Austin BT, Von Korff M (1996). Organizing care for patients with chronic illness. Milbank Q.

[5] Wagner EH (1998). Chronic disease management: what will it take to improve care for chronic illness?. Eff Clin Pract.

[6] (2002). Global Report.

[7] Barr VJ, Robinson S, Marin-Link B, Underhill L, Dotts A, Ravensdale D, Salivaras S (2003). The expanded Chronic Care Model: an integration of concepts and strategies from population health promotion and the Chronic Care Model. Hosp Q.

[8] de Bruin SR, Versnel N, Lemmens LC, Molema CC, Schellevis FG, Nijpels G, Baan CA (2012). Comprehensive care programs for patients with multiple chronic conditions: a systematic literature review. Health Policy.

[9] Kaufman ND, Woodley PD (2011). Self-management support interventions that are clinically linked and technology enabled: can they successfully prevent and treat diabetes?. J Diabetes Sci Technol.

[10] Siminerio LM (2010). The role of technology and the chronic care model. J Diabetes Sci Technol.

[11] Jahns R (2013). The market for mHealth app services will reach $26 billion by 2017.

[12] Nordic Centre for Welfare and Social Issues (2010). Focus on welfare technology.

[13] Grimshaw JM, Thomas RE, MacLennan G, Fraser C, Ramsay CR, Vale L, Whitty P, Eccles MP, Matowe L, Shirran L, Wensing M, Dijkstra R, Donaldson C (2004). Effectiveness and efficiency of guideline dissemination and implementation strategies. Health Technol Assess.

[14] Mair FS, May C, O'Donnell C, Finch T, Sullivan F, Murray E (2012). Factors that promote or inhibit the implementation of e-health systems: an explanatory systematic review. Bull World Health Organ.

[15] Gammon D, Johannessen LK, Sørensen T, Wynn R, Whitten P (2008). An overview and analysis of theories employed in telemedicine studies. A field in search of an identity. Methods Inf Med.

[16] Pearson ML, Wu S, Schaefer J, Bonomi AE, Shortell SM, Mendel PJ, Marsteller JA, Louis TA, Rosen M, Keeler EB (2005). Assessing the implementation of the chronic care model in quality improvement collaboratives. Health Serv Res.

[17] Øvretveit J, Gustafson D (2002). Evaluation of quality improvement programmes. Qual Saf Health Care.

[18] ØVretveit J, Bate P, Cleary P, Cretin S, Gustafson D, McInnes K, McLeod H, Molfenter T, Plsek P, Robert G, Shortell S, Wilson T (2002). Quality collaboratives: lessons from research. Qual Saf Health Care.

[19] Arksey H, O'Malley L (2005). Scoping studies: towards a methodological framework. International Journal of Social Research Methodology.

[20] Levac D, Colquhoun H, O'Brien KK (2010). Scoping studies: advancing the methodology. Implement Sci.

[21] Wagner EH, Davis C, Schaefer J, Von Korff M, Austin B (1999). A survey of leading chronic disease management programs: are they consistent with the literature?. Manag Care Q.

[22] Wagner EH, Austin BT, Davis C, Hindmarsh M, Schaefer J, Bonomi A (2001). Improving chronic illness care: translating evidence into action. Health Aff (Millwood).

[23] Bodenheimer T, Wagner EH, Grumbach K (2002). Improving primary care for patients with chronic illness: the chronic care model, Part 2. JAMA.

[24] Bodenheimer T, Wagner EH, Grumbach K (2002). Improving primary care for patients with chronic illness. JAMA.

[25] Hsieh HF, Shannon SE (2005). Three approaches to qualitative content analysis. Qual Health Res.

[26] Darkins A, Ryan P, Kobb R, Foster L, Edmonson E, Wakefield B, Lancaster AE (2008). Care Coordination/Home Telehealth: the systematic implementation of health informatics, home telehealth, and disease management to support the care of veteran patients with chronic conditions. Telemed J E Health.

[27] Green BB, Cook AJ, Ralston JD, Fishman PA, Catz SL, Carlson J, Carrell D, Tyll L, Larson EB, Thompson RS (2008). Effectiveness of home blood pressure monitoring, Web communication, and pharmacist care on hypertension control: a randomized controlled trial. JAMA.

[28] Gulmans J, Vollenbroek-Hutten M, van Gemert-Pijnen L, van Harten W (2012). A web-based communication system for integrated care in cerebral palsy: experienced contribution to parent-professional communication. Int J Integr Care.

[29] Hess R, Bryce CL, Paone S, Fischer G, McTigue KM, Olshansky E, Zickmund S, Fitzgerald K, Siminerio L (2007). Exploring challenges and potentials of personal health records in diabetes self-management: implementation and initial assessment. Telemed J E Health.

[30] Lyles CR, Harris LT, Le T, Flowers J, Tufano J, Britt D, Hoath J, Hirsch IB, Goldberg HI, Ralston JD (2011). Qualitative evaluation of a mobile phone and web-based collaborative care intervention for patients with type 2 diabetes. Diabetes Technol Ther.

[31] MacLean CD, Littenberg B, Gagnon M (2006). Diabetes decision support: initial experience with the Vermont diabetes information system. Am J Public Health.

[32] Musacchio N, Lovagnini Scher A, Giancaterini A, Pessina L, Salis G, Schivalocchi F, Nicolucci A, Pellegrini F, Rossi MC (2011). Impact of a chronic care model based on patient empowerment on the management of Type 2 diabetes: effects of the SINERGIA programme. Diabet Med.

[33] Nundy S, Dick JJ, Goddu AP, Hogan P, Lu CY, Solomon MC, Bussie A, Chin MH, Peek ME (2012). Using mobile health to support the chronic care model: developing an institutional initiative. Int J Telemed Appl.

[34] Dorr DA, Wilcox A, Donnelly SM, Burns L, Clayton PD (2005). Impact of generalist care managers on patients with diabetes. Health Serv Res.

[35] Roth AM, Ackermann RT, Downs SM, Downs AM, Zillich AJ, Holmes AM, Katz BP, Murray MD, Inui TS (2010). The structure and content of telephonic scripts found useful in a Medicaid Chronic Disease Management Program. Chronic Illn.

[36] Schillinger D, Handley M, Wang F, Hammer H (2009). Effects of self-management support on structure, process, and outcomes among vulnerable patients with diabetes: a three-arm practical clinical trial. Diabetes Care.

[37] Williams GC, Lynch M, Glasgow RE (2007). Computer-assisted intervention improves patient-centered diabetes care by increasing autonomy support. Health Psychol.

[38] Caruso LB, Clough-Gorr KM, Silliman RA (2007). Improving quality of care for urban older people with diabetes mellitus and cardiovascular disease. J Am Geriatr Soc.

[39] Fifield J, McQuillan J, Martin-Peele M, Nazarov V, Apter AJ, Babor T, Burleson J, Cushman R, Hepworth J, Jackson E, Reisine S, Sheehan J, Twiggs J (2010). Improving pediatric asthma control among minority children participating in medicaid: providing practice redesign support to deliver a chronic care model. J Asthma.

[40] Fortney JC, Pyne JM, Edlund MJ, Williams DK, Robinson DE, Mittal D, Henderson KL (2007). A randomized trial of telemedicine-based collaborative care for depression. J Gen Intern Med.

[41] Smith SA, Shah ND, Bryant SC, Christianson TJ, Bjornsen SS, Giesler PD, Krause K, Erwin PJ, Montori VM, Evidens Research Group (2008). Chronic care model and shared care in diabetes: randomized trial of an electronic decision support system. Mayo Clin Proc.

[42] Young AS, Mintz J, Cohen AN (2004). Using information systems to improve care for persons with schizophrenia. Psychiatr Serv.

[43] Chan J, So W, Ko G, Tong P, Yang X, Ma R, Kong A, Wong R, Le Coguiec F, Tamesis B, Wolthers T, Lyubomirsky G, Chow P (2009). The Joint Asia Diabetes Evaluation (JADE) Program: a web-based program to translate evidence to clinical practice in Type 2 diabetes. Diabet Med.

[44] Grimshaw JM, Thomas RE, MacLennan G, Fraser C, Ramsay CR, Vale L, Whitty P, Eccles MP, Matowe L, Shirran L, Wensing M, Dijkstra R, Donaldson C (2004). Effectiveness and efficiency of guideline dissemination and implementation strategies. Health Technol Assess.

[45] Wigfield A, Wright K, Burtney E, Buddery D (2013). Assisted Living Technology in social care: workforce development implications. Jnl of Assistive Technologies.

[46] Samoutis GA, Soteriades ES, Stoffers HE, Philalithis A, Delicha EM, Lionis C (2010). A pilot quality improvement intervention in patients with diabetes and hypertension in primary care settings of Cyprus. Fam Pract.

[47] Morrow RW, Fletcher J, Kelly KF, Shea LA, Spence MM, Sullivan JN, Cerniglia JR, Yang Y (2013). Improving diabetes outcomes using a web-based registry and interactive education: a multisite collaborative approach. J Contin Educ Health Prof.

[48] European Network of Living Labs.

[49] Agency for Healthcare Research and Quality Patient Centered Medical Home Resource Center.

