# Supplementary material for: The Chronic Care Model and Technological Research and Innovation: A Scoping Review at the Crossroads
Source: J Med Internet Res. 2015 Feb 6;17(2):e25. doi: 10.2196/jmir.3547 (PMC4342659; doi:10.2196/jmir.3547)
Supplement: Supplementary file 1 [file jmir_v17i2e25_app1.pdf]

Multimedia Appendix 1.

Search words used
"Chronic Care Model" and "Internet"<br>"Chronic Care Model" and "telemedicine"<br>"Chronic Care Model" and "information and communication technology"<br>"Chronic Care Model" and "ICT"<br>"Chronic Care Model" and "e-health"<br>"Chronic Care Model" and "electronic health"<br>"Chronic Care Model" and "health informatics"<br>"Chronic Care Model" and "interactive health communication applications"
